# Technological Convergence: Highlighting the Power of CRISPR Single-Cell Perturbation Toolkit for Functional Interrogation of Enhancers

**DOI:** 10.3390/cancers15143566

**Published:** 2023-07-11

**Authors:** Reza Ghamsari, Joseph Rosenbluh, A Vipin Menon, Nigel H. Lovell, Hamid Alinejad-Rokny

**Affiliations:** 1 BioMedical Machine Learning Lab (BML), The Graduate School of Biomedical Engineering, UNSW Sydney, Sydney, NSW 2052, Australia; 2 Epigenetics and Development Division, The Walter and Eliza Hall Institute of Medical Research, Parkville, VIC 3052, Australia; 3 Department of Biochemistry and Molecular Biology, Biomedicine Discovery Institute, Monash University, Melbourne, VIC 3800, Australia; sefi.rosenbluh@monash.edu; 4 The Graduate School of Biomedical Engineering, UNSW Sydney, Sydney, NSW 2052, Australia; 5 Tyree Institute of Health Engineering (IHealthE), UNSW Sydney, Sydney, NSW 2052, Australia; 6 UNSW Data Science Hub, UNSW Sydney, Sydney, NSW 2052, Australia

**Keywords:** single-cell perturbation, enhancer, CRISPR, crisprQTL, epigenome

## Abstract

**Simple Summary:**

Enhancers serve as logic gates of the regulatory mechanism of gene expression, and their malfunction is associated with numerous diseases. Therefore, the functional validation of enhancer elements is of great importance in genomics research. Recent technological advancements have enabled the perturbation of enhancers and the examination of their impact on the expression of nearby genes. Here, we review the progress made in experimental and computational methods, which have equipped researchers with a promising arsenal to uncover relationships between enhancers and phenotypes, providing mechanistic insights into diseases.

**Abstract:**

Higher eukaryotic enhancers, as a major class of regulatory elements, play a crucial role in the regulation of gene expression. Over the last decade, the development of sequencing technologies has flooded researchers with transcriptome-phenotype data alongside emerging candidate regulatory elements. Since most methods can only provide hints about enhancer function, there have been attempts to develop experimental and computational approaches that can bridge the gap in the causal relationship between regulatory regions and phenotypes. The coupling of two state-of-the-art technologies, also referred to as crisprQTL, has emerged as a promising high-throughput toolkit for addressing this question. This review provides an overview of the importance of studying enhancers, the core molecular foundation of crisprQTL, and recent studies utilizing crisprQTL to interrogate enhancer-phenotype correlations. Additionally, we discuss computational methods currently employed for crisprQTL data analysis. We conclude by pointing out common challenges, making recommendations, and looking at future prospects, with the aim of providing researchers with an overview of crisprQTL as an important toolkit for studying enhancers.

## 1. Introduction

The human genome contains about 19,000 protein-coding genes, which make up less than 1–2% of the genome. The expression of these genes is regulated by DNA elements that occupy the remaining part of our DNA, making up approximately 99% and known as ‘dark matter’. It is estimated that about 15 million transcription factor binding sites are located in more than 3 million regulatory DNA regions [1]. Understanding the mechanisms of gene expression regulation in cells is a necessity and is at the forefront of genomics research. Non-coding regions play a crucial role in gene expression regulation, and it is estimated that more than 90% of disease- and trait-associated variants fall in these regions [2,3].

Our fascination with enhancers dates back to the 1980s [4]. Enhancers are non-coding sequences and a major class of cis-regulatory elements. They can regulate gene expression independent of their distances and orientation to the transcription starting site when bound by transcription factors [5]. Many causal non-coding loci, identified by genome-wide association studies (GWAS) that are associated with human diseases, are located in enhancer regions [6]. The contributions of single-nucleotide polymorphisms (SNPs) located in enhancers to several diseases, developmental disorders, tumorigenesis, and cancers, underscore the therapeutic potential of enhancers [7].

The Encyclopaedia of DNA Elements (ENCODE) project and the Roadmap Epigenomics Program have identified key features and characteristics of active enhancers, revealing a significant view of the global map of regulatory elements [8,9]. Despite a large body of work focused on enhancer prediction through biochemical and structural tools such as profiling of chromatin accessibility and histone marks, distinguishing between functional and non-functional enhancers remains one of the greatest challenges in the field. Besides the large distance between enhancers and their target genes, the cell-type-specific gene regulation makes predicting their target genes challenging. Advances in large-scale parallel perturbations of enhancers allow for analysis of enhancer–phenotype correlations and the proposal of causal relationships. Inspired by the research surrounding the expression-quantitative trait loci (eQTL), Gasperini et al. developed an experimental strategy, termed crisprQTL. In an analogous manner, they replaced individuals, variants, and tissue-level RNA sequencing in eQTL with cells, various combinations of sgRNAs, and single-cell RNA sequencing (scRNAseq) readouts in crisprQTL, respectively [10].

This review highlights the use of crisprQTL as a versatile toolkit for dissecting enhancer functions.

## 2. A High-Throughput Toolkit for Precision Epigenome Editing

The emergence of precision editing tools targeting the epigenome, without altering the genome, provides a versatile approach in interrogating causal relationships between the epigenome and transcriptome [11]. Different DNA-binding proteins such as zinc finger proteins (ZFPs), transcription-activator-like effectors (TALEs), and clustered regularly interspaced short palindromic repeats (CRISPR)/nuclease-deactivated Cas9 (dCas9), have been employed to target fused epigenetic modifiers to specific loci in the genome. Among these, dCas9 offers a robust adjustable DNA recognition mechanism without altering the underlying DNA sequence [12].

CRISPR was first discovered in the adaptive immune systems of bacteria and has advanced our understanding of gene function [13]. The CRISPR-Cas9 system consists of two main components: a Cas9 endonuclease and a guide RNA molecule, the latter directing the first component to a specific genomic location. Deactivated Cas9 coupled with various activators or repressors has been employed to investigate enhancer function. Compared to Cas9, dCas9 does not cut the DNA, but can be linked to chromatin modifiers such as Krüppel-associated box (KRAB) to induce chromatin changes [14,15]. The fusion of KRAB, a transcriptional repressor domain, with dCas9 equipped researchers with an incredibly powerful tool known as CRISPR interference (CRISPRi) [16]. A single-guide RNA (sgRNA) directs dCas-KRAB to the genomic location, wherein KRAB induces a heterochromatin state of DNA, thereby promoting histone methylation and deacetylation. The precise epigenome editing capability of the dCas9-KRAB platform, enables the targeting of candidate enhancers in a highly specific manner [17]. A recent study has identified a novel putative enhancer located upstream of colony-stimulating factor 1 (CSF1), a highly expressed gene in triple-negative breast cancer cells. They used CRISPRi assays (dCas9-KRAB) followed by bulk RNA sequencing to validate the contribution of this enhancer to CSF1 expression [18]. Despite the capability of this precise perturbation in enhancing understanding of the correlation between enhancers and phenotypes, the need for a more high-throughput toolkit becomes evident when investigating multiple perturbations within cell type and state-specific scope.

The introduction of scRNA-seq has significantly deepened the understanding of individual cells. Although single-cell technologies provide unprecedented descriptive information, they should be combined with other functional genomics tools to study causality of genes [19]. By combining CRISPRi and scRNA-seq toolkits, Perturb-seq and CRISP-seq have equipped scientists with new powerful tools to investigate and explore genomic element interactions. In these methods, sgRNA was not directly detectable via scRNA-seq due to the lack of a poly (A) tail [20,21,22]. However, by including the sgRNA at the 3’ end of a polyadenylated mRNA transcript, CROP-seq made the direct sequencing of sgRNAs possible and further simplified the construction of sgRNA libraries [23].

One such approach, ‘direct-capture Perturb-seq’, implemented a hybridization-based target enrichment strategy, offered a flexible guide design and targeted sequencing in a cost-efficient way. The 5’ and 3’ platforms of this method were commercialized by 10× Genomics [24]. By integrating direct-capture Perturb-seq with CRISPR gene-tiling screen, Sc-Tiling was developed to investigate the functional domain of a gene at a sub-gene resolution [25]. In another study, expanded CRISPR-compatible cellular indexing of transcriptomes and epitopes by sequencing (ECCITE-seq) combined crisprQTL with cell surface antigen readouts to detect several modalities at a single-cell resolution. Given that protein detection is more sensitive than mRNA, ECCITE-seq, by detecting both modalities, can provide a more robust characterization of cellular phenotypes in response to perturbations. This can be particularly useful in studying heterogeneous cell populations in diseases like cancer [26]. Later, ECCITE-seq was applied to identify new regulatory genes involved in the differentiation mechanism of acute myeloid leukemia [27]. In another study, CRISPR–sciATAC was developed to capture changes in chromatin states following single-cell perturbation screens. This method can be applied to investigate the interplay between specific genetic variants and the chromatin accessibility profile, which can ultimately provide valuable insight into the underlying mechanisms of phenotypes and diseases [28]. Linking genotypes to the phenotype of individual cells has been achieved through the integration of CRISPR perturbations with image-based phenotyping in optical pooled screens. This approach facilitates the measurement of phenotypic features such as cellular morphology, cell–cell interactions, and the localization of molecules at single-cell resolution [29].

**Figure 1:**
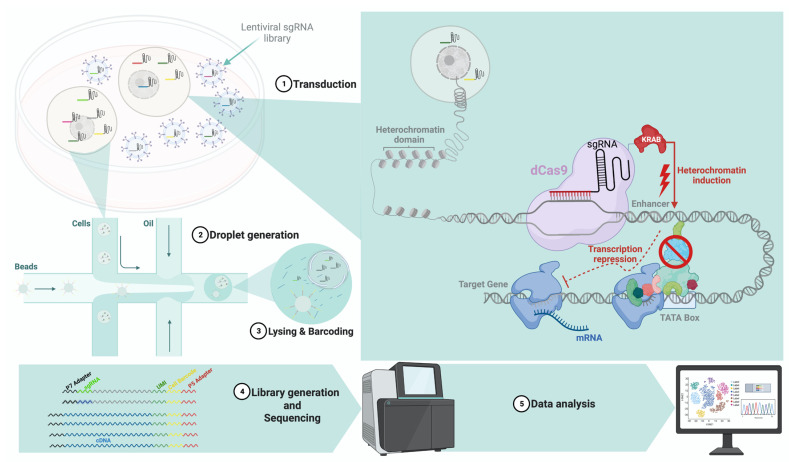
The overall workflow of the crisprQTL toolkit: (1) Transduction: lentiviral delivery of sgRNAs library designed to target candidate regions and the generation of stable cell lines at high multiplicity of infection (MOI). (2) Droplet generation: after screening and selection for the phenotype of interest, polyclonal cells go through droplet generation. (3) Lysing and barcoding: cell lysis is followed by reverse transcription and barcoding. (4) Library generation: pooling samples is followed by PCR to prepare the library ahead of sequencing. (5) Data analysis: data processing, mapping, and assigning sgRNAs to cells.

The use of crisprQTL to alter a large number of enhancers (pooled screens) in a large population of cells (Figure 1) serves as an efficient and high-throughput platform for the interrogation and validation of the function of enhancers in their native genomic contexts.

## 3. Enhancer Interrogation via crisprQTL

The pace of utilizing crisprQTL in functional genomics research has been astonishing, and so far, several research studies have been exploiting this toolkit to functionally explore enhancers. In one study, Mosaic-seq was employed to systematically perturb enhancers and to analyze their activity in both individual and combinational manners. Xie et al. showed that perturbing an enhancer has as significant an impact on gene expression, as targeting the gene itself. They also found that while only a few enhancers within each large cluster of enhancers, also known as super-enhancers, play a major role in repression, perturbing multiple weak enhancers can also change the expression of the target gene [30]. Later, they introduced Mosaic-seq2, which was optimized for the 10× Genomics platform, and also adopted polyadenylation from the CROP-seq method, as mentioned above [31].

In another study, this team optimized their method by increasing the number of utilized sgRNAs per enhancer and performing a gene-specific *p*-value correction. They used this method to identify the secondary target genes regulated by enhancers and to construct an enhancer-driven gene regulatory network. They showed that targeting an enhancer that regulates a transcription factor can result in downregulation of multiple secondary targets [32].

In a separate study conducted by Gasperini et al., crisprQTL mapping was utilized to investigate the presence of multiple sgRNAs within each individual cell. By avoiding an assumption about the target gene, crisprQTL avoids the limitations of gene-specific assays. They showed that introducing sgRNA at a high multiplicity of infection (even to ~28) within individual cells does not reduce the power of CRISPRi [10].

CROP-seq-based methods have certain drawbacks when it comes to genes with a low level of gene expression. Targeted Perturb-seq (TAP-seq) has increased the sensitivity of these approaches by amplifying only a limited number of genes of interest ahead of sequencing, thereby reducing sequencing requirements. TAP-seq not only increases the sensitivity of capturing low-level RNA transcripts but also increases the efficiency of retrieving the gRNA identity. This method also addresses the multiple hypothesis testing problems by focusing on a predefined set of genes and targeted readout of their relevant transcripts [33]. In another study, systematic targeting and inhibition of non-coding GWAS loci with single-cell sequencing (STING-seq) utilized crisprQTL to study gene-regulatory functions of multiple GWAS variants. This approach connects GWAS variants with their respective target genes, enabling high-throughput exploration of relationships between genetic variants and their functional outcomes, and holds a potential to discover therapeutic targets [34].

A recent study by Armendariz et al. employed crisprQTL to investigate the contribution of enhancers to developmental diseases by examining how they alter cell fate determination. They functionally interrogated 25 enhancers associated with congenital heart defects in stem cells, ahead of their differentiation to cardiomyocytes. They measured the transcriptome and found a subset of 16 enhancers where the perturbation caused a delay in the specification of human cardiomyocytes [35]. In another study, a multi-assay evaluation of GWAS target genes was carried out using functional CRISPR screens, High-throughput Chromosome Conformation Capture with Chromatin Immunoprecipitation (HiChIP), and crisprQTL to evaluate the relationship between candidate breast cancer-associated enhancers and their target genes [36].

Most recently, crisprQTL has been implemented in a comprehensive study, which focused on regions containing mutations associated with psychiatric disorders, perturbing almost one thousand enhancers in primary human astrocytes. They found almost one hundred active enhancers that contain disease-associated variants and additionally pinpointed 140 enhancer target genes linked to these disorders [37].

To date, most functional studies have focused on unraveling the mystery of gene regulation by perturbing enhancers at a DNA level. However, a recent study introduced Cas13 RNA Perturb-seq (CaRPool-seq) that can perform multiplexed combinatorial perturbations by targeting enhancer RNAs [38].

## 4. Computational and Statistical Toolkits

In parallel with the development of the experimental procedures of crisprQTL, there is an ongoing effort to develop computational methods to assist downstream analysis and improve the interpretation of generated data.

Large-scale functional studies have been conducted to evaluate the efficiency of the Cas system as a toolkit for functional genomics [39,40,41,42]. However, the design of sgRNA has been a major bottleneck across these studies, given its crucial role in determining the success of knockouts or knockdowns. To address this issue, several computational models, based on these functional studies, have been developed with the aim to identify design principles for highly effective sgRNA [43,44,45,46]. These models incorporate state-of-the-art machine learning [43,46] and deep learning [47,48] models to identify biological features that influence the sgRNA efficiency. This research has led to the development of high-performing libraries of sgRNA [46]. Both experimental and computational studies have shown that features related to the structural stability and flexibility of sgRNA, such as GC content and self-folding energy significantly contribute to the efficiency of the CRISPR system [43,44]. Tools are available online and via the command line for scoring highly effective sgRNA for any given library.

For the downstream analyses, a few public datasets exist for functional analysis of enhancers using crisprQTL. In two early reported datasets, different statistical methods were applied to analyze differential expression. In the first one, Xie et al. [30] used nonparametric test for independence, Chi-squared-like tests, which had limitations in dealing with technical confounders. In the second one, Gasperini et al. [10] applied negative binomial general linear models (GLMs), which were not robust to the misspecification of the gene expression model. These two datasets were later reanalyzed with SCEPTRE and GLM-EIV. SCEPTRE implemented a form of conditional randomization test to address the miscalibration problem highlighted in the two aforementioned studies. By examining negative control sgRNAs, SCEPTRE provided a reassessment of the calibration of these methods. Upon applying SCEPTRE, a new set of enhancers (>40) was introduced to Gasperini’s dataset, while a set of introduced enhancers (>20) in the original research was removed [49]. SCEPTRE demonstrates the sensitivity and specificity of crisprQTL and addresses the previously mentioned limitations. However, like other standard analysis methods, SCEPTRE imputes perturbation assignments onto cells by applying the thresholded regression method on the sgRNA counts and assumes away measured noise. In another attempt, a method known as GLM-based errors-in-variables (GLM-EIV), used a new class of measurement error models to select a threshold and estimate the probability of perturbation in each cell, as well as the effect size of it on differential gene expression. GLM-EIV was applied to both datasets and outperformed the thresholded regression method in settings with high background contamination, where an sgRNA incorrectly assigned to a cell [50].

In another computational approach, the single-cell model-based analysis of genome-wide CRISPR/Cas9 knockout (scMAGeCK) utilized the negative binomial distribution, a generalized linear model, and expectation maximization approach to model perturbation. This approach introduced two different modules. (1) The first module can detect enhancers linked to only one single target gene, based on the robust rank aggregation algorithm. (2) The second module can assess the impact of perturbation on thousands of target genes based on linear regression [51].

In a different study, a computational pipeline was developed to remove the clonal cell expansion effect in crisprQTL experiments, leading to significant reduction of false discoveries. This pipeline leverages the combination of multiple sgRNAs in each cell, using them as a barcode to identify distinct clones [52]. In another separate study, GLiMMIRS was developed using negative binomial generalized linear models, accounting for sgRNA efficiency and several other covariates. Reanalysis of Gasperini’s dataset [10] using this method, which investigated interactions between 3,808 enhancer pairs, showed that enhancers act in a multiplicative manner; however, the results provided no evidence for strong interactions between pairs of enhancers [53]. Most recently, SCREE has been introduced as a comprehensive workflow that facilitates crisprQTL data analysis. This flexible pipeline offers a platform for performing pre-processing and downstream analysis of RNA sequencing data, assay for transposase-accessible chromatin with sequencing (ATAC-seq) data, and multimodal 10x-based readout. It is implemented in open-source Python and R packages, and is accompanied by a tutorial [54].

## 5. Pitfalls and Recommendations

The experimental design of a pooled CRISPR screen was comprehensively described in a recent review [55]. Despite the great promise of crisprQTL, a plethora of variables may affect the experiment and should be considered prior to the experiment design and during the data analysis. The number of perturbations in each cell depends on the number of sgRNA stochastically introduced into that cell [33]. One of the primary stages of the experiment is designing optimal sgRNA. However, the design is limited by requirements of the protospacer adjacent motif (PAM), which restricts the number of target regions, including those involved in gene regulation. Another limitation is the trade-off between efficiency and specificity; sgRNAs with high specificity may have lower efficiency, whereas sgRNAs with high efficiency may have a higher likelihood of off-target effects. Computational prediction of off-target candidate regions can help overcome this limitation [56,57]. Recently, significant progress has been made in both experiment and computational methods to overcome these limitations. A recent study presented high-performance computational models for assessing the impact of sgRNA on different targets across Cas9 variants and orthologs. The study generated high-throughput datasets for all Cas9 variants [58] and orthologs [59], and identified relevant biological features, such as GC content, melting temperature, and self-folding energy of spacer sequence, that influence sgRNA activity. The paper provides a web tool link for researchers to utilize variants and orthologs based on experiment needs. Although the current designs are highly efficient, further improvements can be achieved by integrating the structural features of both the spacer and scaffold of sgRNA. Previous research has suggested that altering the secondary structure of sgRNA through engineering sequences within tetra loop regions improves editing efficiency [60]. For assessing off-target effects, both heuristic and machine-learning-based methods have been developed, which could be incorporated to identify highly active and specific sgRNAs. However, they currently work only for the human genome and, to some extent, for mice. Assessments on major model organisms like drosophila, zebrafish, and C. elegans, have yet to be achieved [56,61]. In the context of CRISPR multiplexing perturbations, the competition of several sgRNAs for endonucleases in a cell, known as retroactive effects, can change the efficacy of every sgRNA [62]. A focused validation, through targeting enhancers by an active Cas9 protein, can also help to balance speed with accuracy [35].

KRAB induces histone deacetylation, H3K9 methylation and a heterochromatin domain formation. During an investigation of enhancers located more than 1 kb distant from the transcription start site, it was observed that the KRAB effect does not spread toward promoter linearly along the DNA [33]. Moreover, due to three-dimensional interactions in the genome, KRAB can induce heterochromatin formation in a non-linear manner [17]. Therefore, the repression of the putative gene might be induced by KRAB rather than the enhancer itself. The extent of spreading of the KRAB effect within DNA depends on different factors, including time, and can range from 1 kb to 200 kb. Other factors, such as the level of activation, genomic locus, and neighboring elements like insulators, enhancers, and promoters can also influence this spreading [63]. Considering the time dependency of enhancer functions, a delay in the KRAB function could pose a technical challenge while sgRNAs are introduced at a fixed time point [35].

The interaction between neighboring cells can confound gene expression and should be taken into account [64]. Gene expression can be regulated by multiple enhancers, which may mask the effect of perturbation. Moreover, the activity of enhancers and their contribution to gene expression are heterogeneous across a cell population. It is also important to note that enhancers can compensate for one another, therefore sometimes targeting multiple weak enhancers among some super-enhancers is necessary to repress gene expression [30,65].

As discussed above, despite the high efficiency of CRISPRi for perturbation across a cell population, it is susceptible to incomplete inhibition. The variability in sgRNA targeting efficacy and dCas9-KRAB perturbation should be considered [10], and GLiMMIRS uses a statistical model to account for this covariate [53].

While a high ratio of a lentiviral transduction to the number of target cells, known as the multiplicity of infection (MOI), can improve the sensitivity and specificity of the screen [51], an excessively high MOI could also affect the physiological response of the cell. Therefore, it is important to consider the MOI tolerance threshold for different cell types, especially when most sgRNAs have an effect. Thus, it might be necessary to carefully optimize and calibrate the MOI tolerance. Multiple perturbations in a cell can either facilitate or impede the cell proliferation rate and trigger a clonal expansion, introducing a potential bias in sampling. By considering the clonal expansion artifacts during the experimental design and applying computational methods to filter out clonal cells, biases, and false discoveries can be minimized [52].

From a technical perspective, scRNA-seq data is intrinsically noisy data. Various studies have addressed data correction and noise reduction [66]. A low amount of start material can affect the success of screening, particularly for genes with low expression levels. Targeted sequencing or high-content read-outs can address this issue, although the latter is limited by sequencing costs [33]. Simultaneous profiling of gene expression and other modalities within the same cell, such as multiome [67] and CITE-seq [26], offer potential solutions to overcome the drop-out limitation associated with the low abundance of transcripts.

Finally, to avoid unintended interferences and biases, it is crucial to consider and evaluate the influencing variables mentioned above prior to, during, and after the experimental process, throughout the data analysis.

## 6. Conclusions and Future Prospective

The crisprQTL toolkit has enabled a facile parallel perturbation of a large number of enhancers and the analysis of the resulting phenotype in each cellular comportment. We highlighted several recent research studies, and collectively, these studies have demonstrated the potential of crisprQTL in investigating the correlation between enhancers and their target genes. With an ever-growing generation of epigenomic data, such as enhancerRNA expression, high-throughput chromosome conformation capture (Hi-C), ATAC-seq, and chromatin immunoprecipitation, followed by sequencing (ChIP-seq), introducing more candidate enhancers, the functional testing of enhancers will be indispensable. Astonishing progress has been made in single-cell multi-omics approaches, specifically the commercial multi-omics product 10x Multiome [67], which provides an unprecedented opportunity to profile the gene expression and chromatin accessibility within the same cell. Recruiting multiome instead of scRNA with the crisprQTL toolkit can multiply the power of enhancer interrogation. In the future, the incorporation of long-read sequencing and computational tools such as FLAMES (full-length alternative splicing quantification) [68] into the crisprQTL toolkit could also lead to significant advances in our understanding of enhancer roles in splicing and regulation of isoform usage. Other omics modalities could be integrated into the crisprQTL toolkit, along with advancements in genomics, to significantly deepen our understanding of the way enhancers regulate gene expression. In the near term, this will depend on the development of computational and statistical methods. We envision that crisprQTL, as a promising toolkit, can change the landscape of high-throughput functional epigenomics and play a pivotal role in the comprehensive analysis and interrogation of non-coding regions, which could ultimately pave the way to the discovery of novel therapeutic targets.

## Data Availability

Not applicable.
